# Analysis on Characteristics of ZnO Surface Acoustic Wave with and without Micro-Structures

**DOI:** 10.3390/mi10070434

**Published:** 2019-06-30

**Authors:** Huei-Yu Huang, Hsi-Jen Chiang, Ching-Zong Wu, Yi Lin, Yung-Kang Shen

**Affiliations:** 1 Department of Dentistry, Taipei Medical University-Shuang Ho Hospital, New Taipei City 235, Taiwan; 2 School of Dentistry, College of Oral Medicine, Taipei Medical University, Taipei 11031, Taiwan; 3 Department of Dentistry, Taipei Medical University Hospital, Taipei 110, Taiwan; 4 Department of Dentistry, Lotung Poh-Ai Hospital, Yilan 26546, Taiwan; 5 Department of Business Administration, Takming University of Science and Technology, Taipei 114, Taiwan; 6 School of Dental Technology, College of Oral Medicine, Taipei Medical University, Taipei 110, Taiwan

**Keywords:** surface acoustic wave, ZnO thin film, silicon micromachining, signal response

## Abstract

In this paper, we fabricate a surface acoustic wave (SAW) device with micro-structures on a zinc oxide (ZnO) thin film and measure its signal response. The manufacturing processes of the SAW device include the fabrication of micro-structures of a SAW element and its interdigital transducer by silicon micro-machining and the fabrication of a thin film of ZnO by RF magnetron sputtering. We, then, measure the SAW properties. This research investigates the properties of sputtered thin films for various amounts of O_2_/(Ar + O_2_) using Zn and ZnO targets. Regardless of target, the growth rate of the ZnO thin film decreases as the oxygen content increases. When the SAW is sputtered ZnO thin film using 30% oxygen, the digital signal of the SAW has better performance. The measurement signal of the SAW with micro-structures is similar to that without micro-structures.

## 1. Introduction

Surface acoustic waves (SAWs) can be employed to determine mass variations by the frequency diversification method. Micro-electro-mechanical systems (MEMS), which have attracted considerable attention over the past three decades, are commonly used to generate SAWs. The sensor is a very important element of a MEMS, where physical sensors, chemical sensors, and biosensors are the three types of sensors used. A SAW device can act as a sensor in many fields. A SAW can act as a gas sensor to detect the concentration of oxygen, or as a biosensor to detect micro-particles of different kinds (such as polystyrene) or contribute to facilitated blood analysis. The velocity of a shear wave can be measured by the SAW device. The visco-elastic characterization of the mechanical relaxation of a very thin layer could be resolved using a SAW device. The Rayleigh SAW device can be used in a liquid heating system. A SAW sensor with Pt coated ZnO nanorods as the selective layer has been investigated for hydrogen detection. A high-precision UV detector combining a ZnO nanostructure and a SAW oscillator system has been presented. SAW has also been used to examine the mechanisms by which cells scaffold. A SAW device to detect dimethyl methylphosphonate (DMMP) vapor has been designed. 

Lord Rayleigh [1] first indicated that shear and longitudinal waves were between body and acoustic waves. He found that a surface acoustic wave could be transferred along the surface of an elastic body. A researcher applied aluminum (Al) as interdigital transducer (IDT) fingers on a zinc oxide thin film to produce and transmit a piezo-electric signal [2]. The SAW element has been adopted as a humidity detector. Its active frequency was 50 MHz, and it could detect 0–80% humidity, which was reflected in the signal frequency [3]. LiNbO_3_ was fabricated on a silicon substrate and coated with polyvinylidene fluoride (PVDF) to increase surface sliding on a SAW [4]. A SAW element with 72 pairs of interdigital IDT fingers was fabricated on a (100) silicon wafer by silicon micro-machining. Due to the economic importance of the wine market for many countries, instruments for quality control are very useful for the wine industry and regulatory organizations. The SAW has been applied in order to discriminate Spanish wine coming from different grape varieties and elaboration processes [5]. The 20, 30, and 40 μm interdigital IDT fingers structures of a SAW has been manufactured using a negative SU-8 photoresist on silicon micro-machining, which detected the mixed reaction frequency, at various concentrations, between glycerol and wafer [6]. Gronewold [7] compared the latest developments in the emerging field of SAW sensors. The output signal gave information for pure mass loading, the intrinsic properties of bound materials, or visco-elastic effects (such as structural rearrangements). Peptide nebulization has been optimized for detection using a SAW chip by mass spectrometry and tandem mass spectrometry was conducted on SAW-nebulized peptide ions. The peptide tandem mass spectra were recorded, allowing a sequence to be assigned for the peptide, suggesting that this method has general utility in the field of proteomics [8]. A layered nanostructure (semiconductor + metal) created new possibilities for gas sensing in a SAW sensor with acousto-electric application between the surface wave and the sensor structure [9]. SAW acoustophoresis has become an extremely dynamic and exciting field, which is especially useful for lab-on-a-chip applications, where a compact and non-invasive biomanipulation technique has been highly desired [10]. The research in [11] aimed to provide the reader with a historical basis, routes for more detailed study, and an impression of future directions in the field. A two-step measurement, using copper eletro-deposition for instrument calibration followed by a measurement on the actual biological layer under investigation in a combined SAW/SPR instrument, led to an estimate of water content and layer thickness [12]. The influence of surface structure in terms of SAW biosensor signals has been discussed. Polymer structures were applied onto sensor surfaces, either by a lithography method or by the self-assembly of polystyrene micro-particles [13]. A two-port R-SAW resonator biosensor presented high sensitivity and sub-nanomolar LoD (−296 m^2^·kg^−1^ and 104 pM—or 6.2 ng/mL—for the biotinstreptavidin essay, respectively). Biosensors presented by the authors had a dynamic range potentially compatible with several health- and safety-related assays, among all cancer biomarker detections [14].

In this research, a SAW with micro-structure is fabricated by silicon micro-machining on various target materials (metal or ceramic) and their properties are compared. The properties of the thin films of SAW with oxygen of different amounts in the sputtering process, in terms of surface roughness and crystal array lattice, are discussed. An atomic force microscope (AFM) is applied to determine the surface roughness of zinc oxide (ZnO) thin film with oxygen of various amounts. X-ray diffraction (XRD) is employed to measure the crystalline direction of the ZnO thin film with oxygen of various amounts. Finally, a network analyzer is utilized to measure the SAW performance of the ZnO thin film, with and without micro-structures and for different amounts of oxygen.

## 2. Experimental

### 2.1. SAW Preparation

The surface acoustic wave often uses a piezo-electric substrate, where the positive and negative piezo-electric effects are exploited to receive and propagate the signal. The fabrication process of SAW included the manufacture of a zinc oxide thin film and the fabrication of SAW element micro-structures for signal detection. The silicon substrate was a (100), p-type 4-inch SiO_2_ wafer (Tekstarter, USA). Its thickness was 1 μm. Silicon was applied as the substrate material and, then, the ZnO material was sputtered onto its surface as the piezo-electric material. The RF sputtering machine was developed in the authors’ laboratory. The sputtering targets were 99.99% pure ZnO and Zn.

The input and output interdigital IDT fingers (micro-structures) were located on the surface of the piezo-electric substrate of the surface acoustic wave. Interdigital IDT fingers transfer the electrical signal to mechanical energy and consequently transfer the mechanical energy to an electrical signal, for resonance or to filter radio noise due to their piezo-electric characteristics. Interdigital IDT fingers were manufactured on the piezo-electric substrate. Each of interdigital IDT fingers acted as a transducer. Interdigital IDT fingers can transfer the input signal of RF to a sound wave through the piezo-electric effect; it can transfer a sound wave (input signal) to a RF electrical wave (output signal) through the piezo-electric effect, as well.

**Figure 1:**
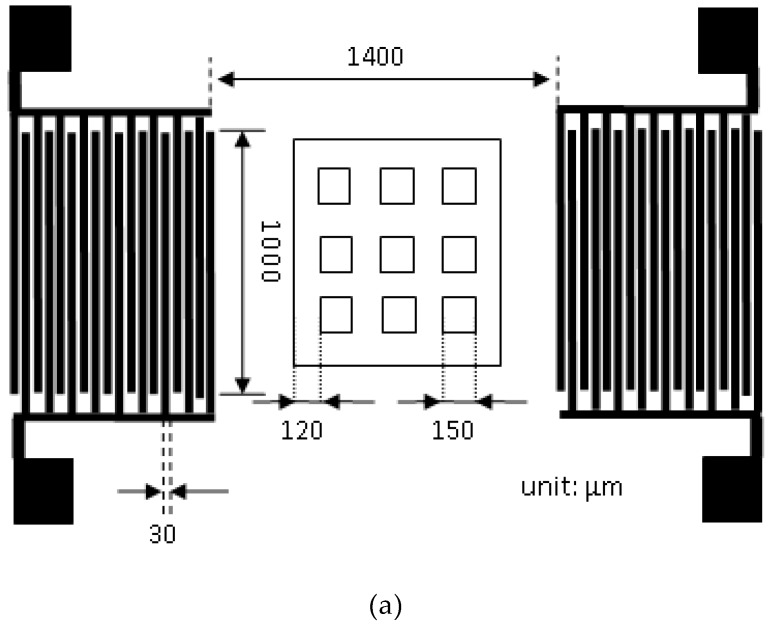

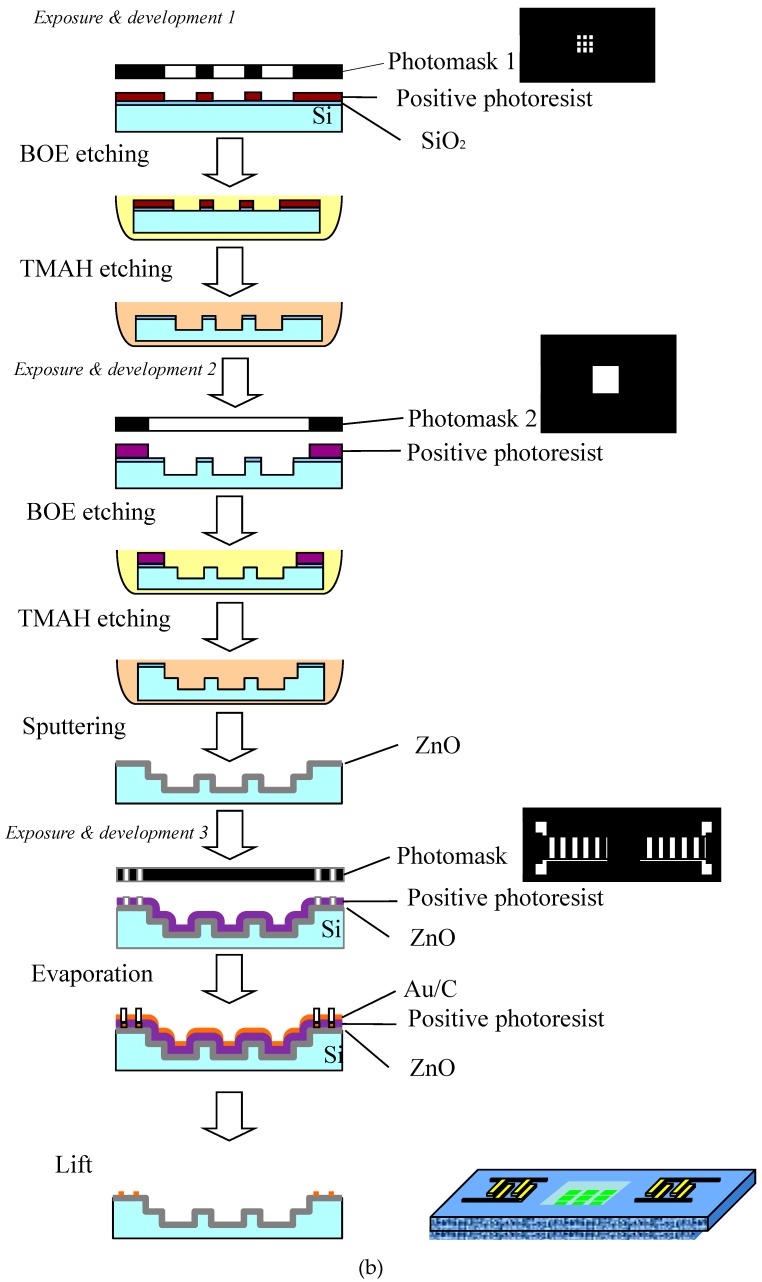
(**a**) The dimensions of a surface acoustic wave (SAW) element. (**b**) The processing flowchart of SAW.

Figure 1a shows the dimensions of the SAW element, where W (interdigital IDT finger overlap length) = 1000 μm, dm (sound wave transmission distance) = 1400 μm, N (interdigital IDT finger pairs) = 20, and d (metal interdigital IDT finger thickness) = 30 μm. Figure 1b displays the processing flowchart of SAW. The silicon wafer with a SiO_2_ layer was cleaned using the standard process. The positive photoresist (AZ-4620, MicroChemicals, Ulm, Germany) was coated onto the surface of silicon wafer using a photoresist coating machine. The positive photoresist was then soft-baked to harden it. The exposure and development method was employed to define the slot hole in the micro-structures of the squares. The micro-structures of the photoresist were then hard-baked to harden it. A buffered oxide etching (BOE) solution was utilized to clean the SiO_2_, whose surface had no photoresist without etching. The first layer of the photoresist was, then, cleaned and immersed in tetramethylammonium hydroxide (TMAH) to perform deeper etching. After the slot hole in the micro-structures was etched, the exposure and development defined the slot hole in the micro-structures using a photomask. BOE was adopted to clean the SiO_2_ layer that was not protected by photoresist. After cleaning, wet etching was performed, using BOE to deep-etch the external slot hole in the micro-structures. An RF sputtering machine was used to sputter the ZnO thin film onto the wafer surface. The sputtering targets used Zn and ZnO materials.

The reactive gas used in the sputtering process was oxygen. The percentages of oxygen used were 10%, 30%, 50%, and 70%. After the ZnO thin film was deposited, the structure of the metal interdigital IDT fingers was defined by a photomask. Exposure and development were conducted. The E-gun evaporator deposited the metal interdigital IDT fingers after the development process. The wafer was immersed in acetone after the deposition process to lift-off the un-needed deposition layer. Gold was used as the evaporation material, due to its high transmission signal power and resistance to oxidation. The thickness of the gold electrode was 2000 Å. As the crystal arrays differ between gold and ZnO, ripping easily occurs upon gold deposition onto ZnO. The process evaporated the Cr material (thickness = 300 Å) as the adhesive layer between the gold and ZnO.

### 2.2. Measurement of SAW (Thickness of Thin Film, Surface Properties, and Frequency)

A surface profiler (Alpha-Step 500, KLA-Tencor, Milpitas, CA, USA) was used to measure the thickness of the deposited thin film. XRD was adopted to inspect the crystal array of thin film and to determine the optimal deposition. SEM (JEM-6500, JEOL, Akishima, Japan) was applied to observe the surface morphology of the thin film. Atomic force microscopy (AFM, DI-3100, Veeco, Plainview, NY, USA) was employed to measure the surface roughness of the thin film. The scanning range of each measurement point was 5 × 5 μm^2^.

**Figure 2:**
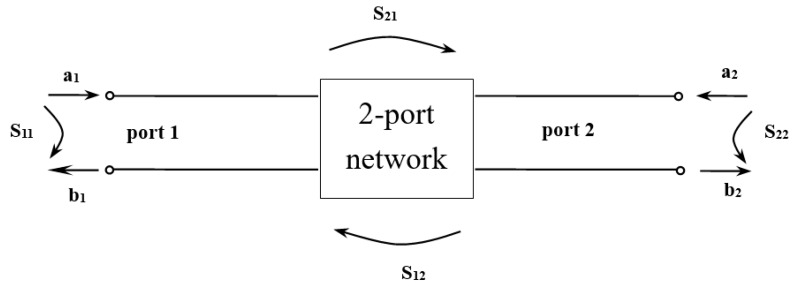
Scattering parameters.

A network analyzer made by the Algelon Company (Taiwan), N5230A PNA-L, was utilized to measure the frequency of the SAW (Figure 2). For a n-port network, the scattering parameter matrix is

(1) $$\left[\begin{array}{l} b_{1}\\ b_{2}\\ b_{3}\\ \vdots \\ b_{n} \end{array}\right]=\left[\begin{array}{c} S_{11}\\ S_{21}\\ \vdots \\ S_{n1} \end{array}\begin{array}{c} S_{12}\\ S_{22}\\ \vdots \\ S_{n2} \end{array}\begin{array}{c} \cdots \\ \cdots \\ \cdots \end{array}\begin{array}{c} S_{1n}\\ S_{2n}\\ \vdots \\ S_{nn} \end{array}\right]\left[\begin{array}{c} a_{1}\\ a_{2}\\ a_{3}\\ \vdots \\ a_{n} \end{array}\right]\;\mathrm{or}\;\left[b\right]={\left[S\right]}^{}\left[a\right],$$

where *a_n_* and *b_n_* are the electrical wave amplitudes of incidence and reflection, respectively, into the *n^th^* port of the *n*-port network. If the network is a 2-port network, the scattering parameter matrix can be simplified to Figure 2. The *S*-parameter can, then, be presented as

(2) $$S_{11}={\frac{b_{1}}{a_{1}}|}_{a_{2}=0}S_{21}={\frac{b_{2}}{a_{1}}|}_{a_{2}=0}S_{22}={\frac{b_{2}}{a_{2}}|}_{a_{1}=0}\;S_{12}={\frac{b_{1}}{a_{2}}|}_{a_{1}=0},$$

where *S*_11_ and *S*_22_ are reflection coefficient, *S*_12_ and *S*_21_ are transmission coefficient. Additionally,

(3) $$RL=20\log\left(\frac{a_{1}}{b_{1}}\right)=20\log\left|\frac{1}{S_{11}}\right|$$

(4) $$IL=\alpha =20\log\left(\frac{a_{1}}{b_{2}}\right)=20\log\left|\frac{1}{S_{21}}\right|,$$

where *RL* is the return loss and *IL* is the insertion loss.

## 3. Results and Discussion

### 3.1. Thin Film Thickness

**Figure 3:**
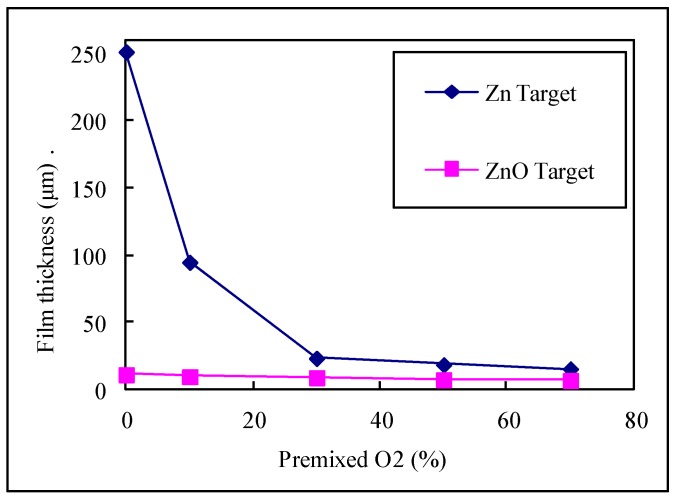
The deposition thickness of ZnO thin film on various targets (substrate temperature: 200℃, residual pressure: 5.0 × 10^−6^ torr, RF power: 100 W).

The relationship between thin film thickness and the percentage of oxygen is elucidated in this research. A surface profiler (α-step 500) was utilized to measure the thickness of the thin film at the position of 2/3 radius of the 4 inch wafer after sputtering. In this investigation, multiple measurements were made at the same position, and the average value of the growth rate of the thin film was determined. Figure 3 shows the deposition thickness of the ZnO thin film with various target materials for different amounts of oxygen (the temperature of substrate was 200 °C, the residual pressure was 5.0 × 10^−6^ torr, and the RF power was 100 W). The results demonstrate that the thin film thickness decreased as the amount of pre-mixed oxygen increased for the various target materials (Zn, ZnO). The results also reveal that the thin film thickness decreased rapidly as the amount of pre-mixed oxygen increased (between 10–30%) for a Zn target. The thin film thickness decreased slightly as the amount of pre-mixed oxygen increased for a ZnO target. The Zn target material had a greater effect on the deposition thickness of the ZnO thin film.

### 3.2. Surface Morphology of the ZnO Thin Film

**Figure 4:**
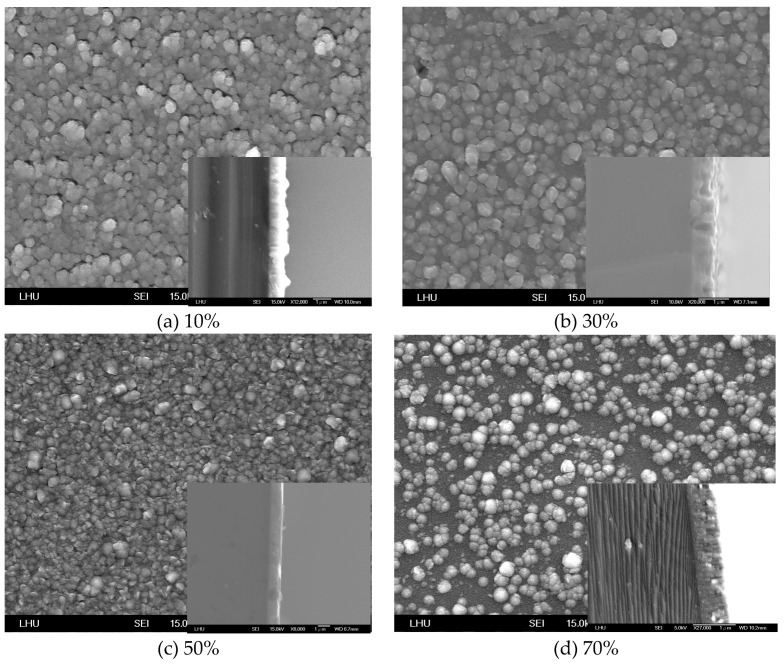
SEM image of the ZnO thin film for various amounts of oxygen with a Zn target.

**Figure 5:**
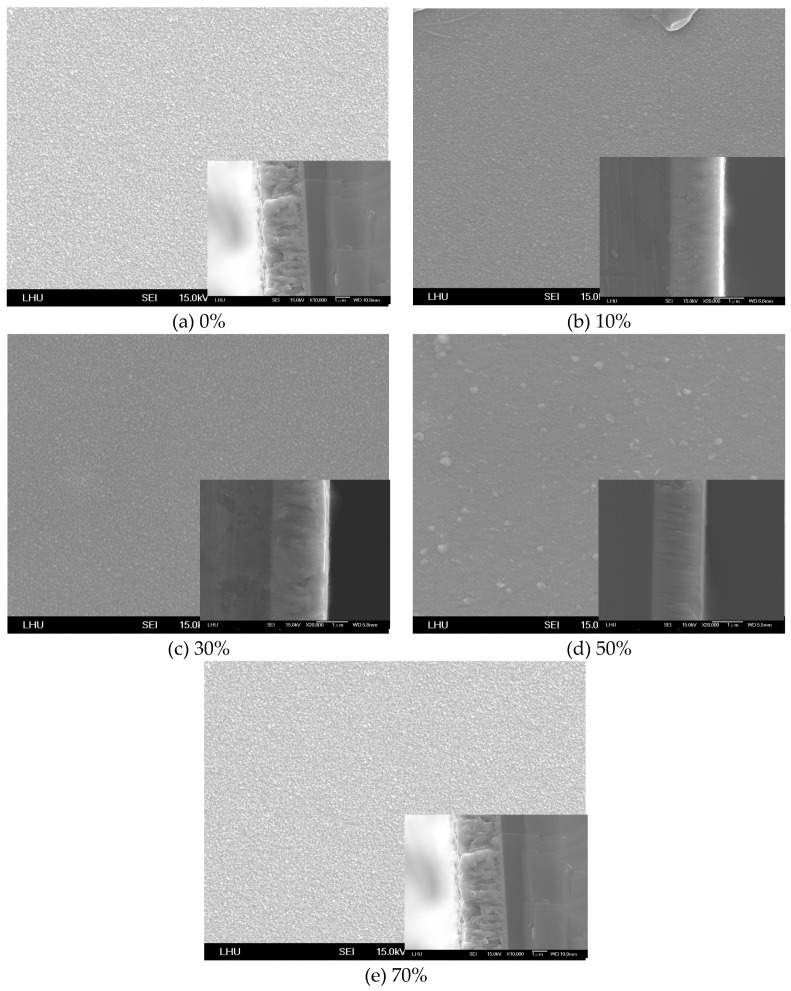
SEM image of the ZnO thin film for various amounts of oxygen with a ZnO target.

In the propagation theory of surface waves, the signal is transferred by surface propagation. The surface flatness of a thin film affects the propagation velocity of sound wave. The surface roughness and crystal array of the ZnO thin film surface were determined. Figure 4 and Figure 5 show the SEM image of the ZnO thin film surface morphology with various amounts of oxygen and for Zn and ZnO targets, respectively. The vignette in the lower right corner of each image in Figure 4 and Figure 5 is the morphology image of the thin film thickness. The results reveal that the crystal particles of the deposition thin film had the smallest value when the amount of oxygen exceeded 50% on a Zn target (Figure 4c). Figure 4c also indicates that the surface flatness of the ZnO thin film for the amount of oxygen exceeding 50% was better than those for 10% and 30% oxygen. The results reveal that larger particles were present on the surface of the ZnO thin film in Figure 4a,d. This phenomenon shows that a larger deposited particle has a higher kinetic energy and higher surface propagation energy, inducing reactions and diffusion on the substrate. Larger crystal particles were formed because the plasma particle induces inelastic collisions. This situation causes particle energy loss, causing it to have insufficient energy to propagate on the surface of the thin film. The surface flatness of the ZnO thin film increased as the amount of oxygen increased in the ZnO target (Figure 5). The results also reveal that the morphology of thickness of the ZnO thin film presented an irregular shape for the Zn target. In contrast, the morphology of thickness of the ZnO thin film appears to have been a uniform growth situation for the ZnO target. The ZnO thin film had the best surface flatness at a 50% oxygen amount and the ZnO target.

### 3.3. Surface Roughness

**Figure 6:**
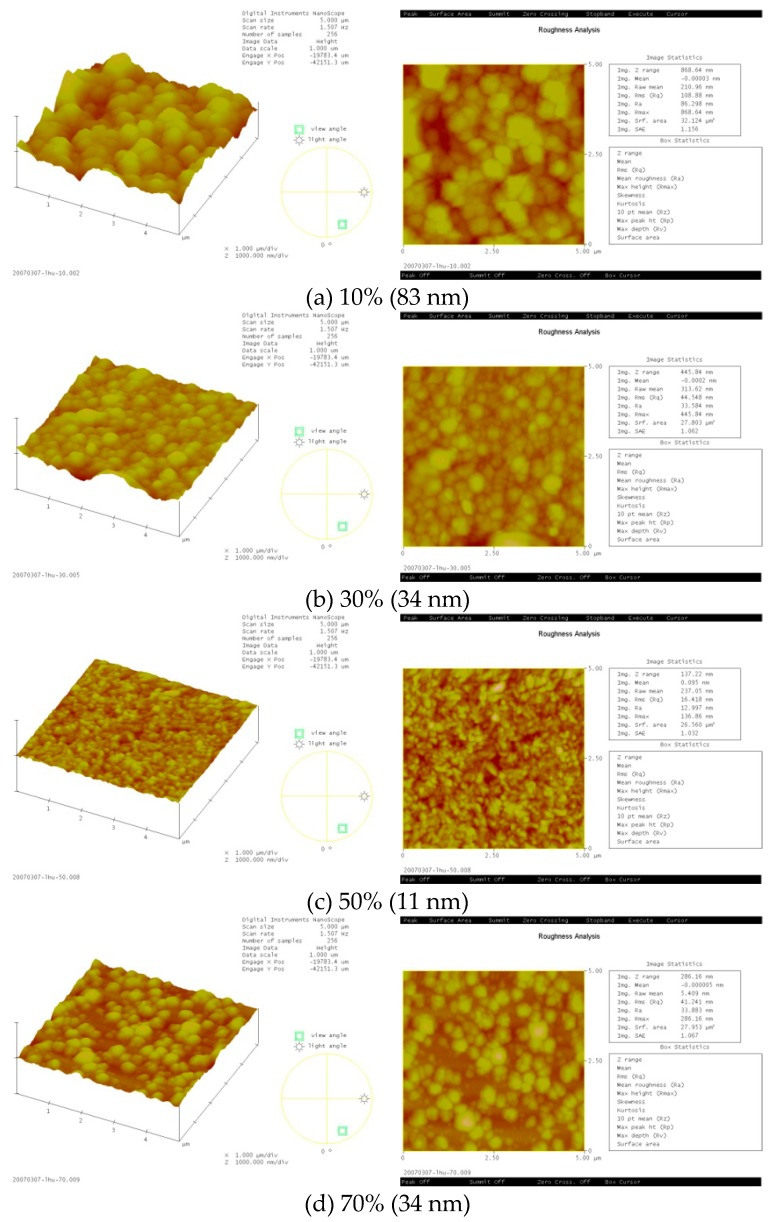
Surface roughness of the ZnO thin film for various amounts of oxygen with a Zn target.

**Figure 7:**
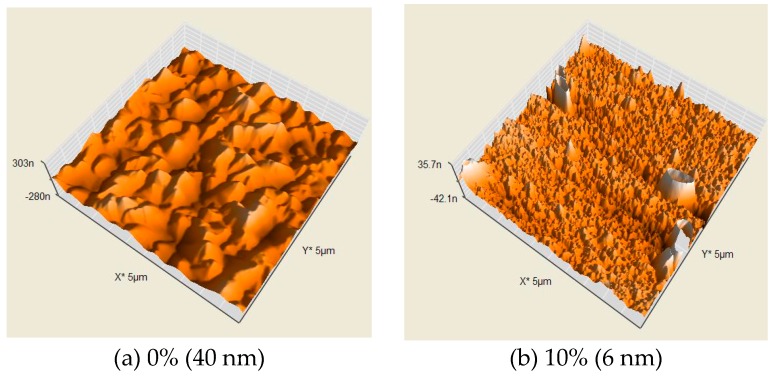

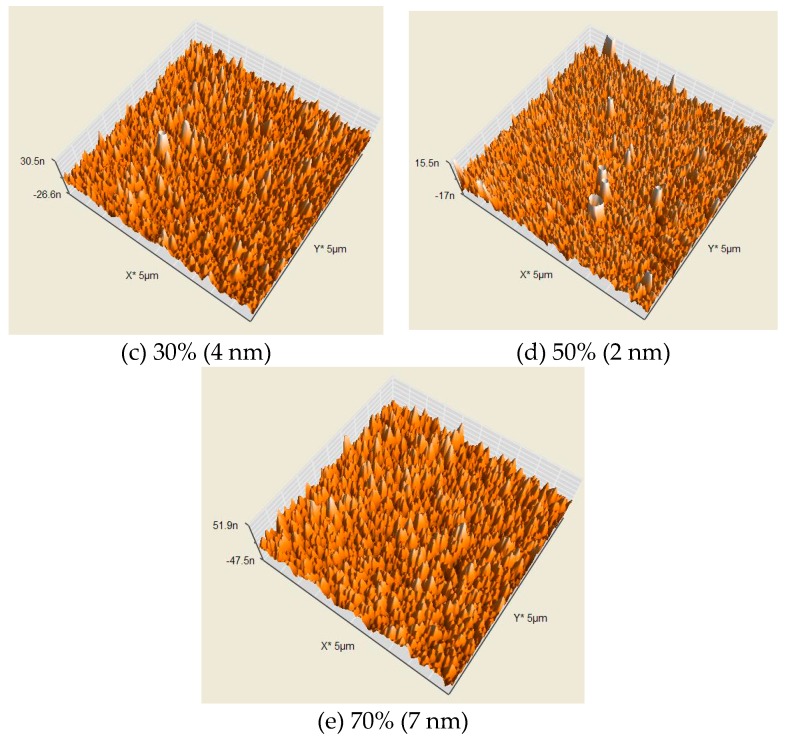
Surface roughness of the ZnO thin film for various amounts of oxygen with a ZnO target.

Figure 6 and Figure 7 display the surface roughness of the ZnO thin film with various amounts of oxygen and either a Zn or ZnO target. The surface roughness of the ZnO thin film decreased as the amount of oxygen increased for the Zn target (10–50%) and increased with an amount of oxygen over 50%. The results demonstrate that the surface roughness of the ZnO thin film was 11 nm (minimum) when the amount of oxygen was 50% for the Zn target. The surface roughness of the ZnO thin film decreased as the amounts of oxygen increased for the ZnO target (0–50%) and increased when the amount of oxygen was over 70%. The results reveal that the surface roughness of ZnO thin film was 2 nm (minimum) when 50% oxygen was used for the ZnO target. The surface roughness of the ZnO thin film obtained a minimum value when 50% oxygen was used, regardless of target.

### 3.4. Lattice of Crystal Array

**Figure 8:**
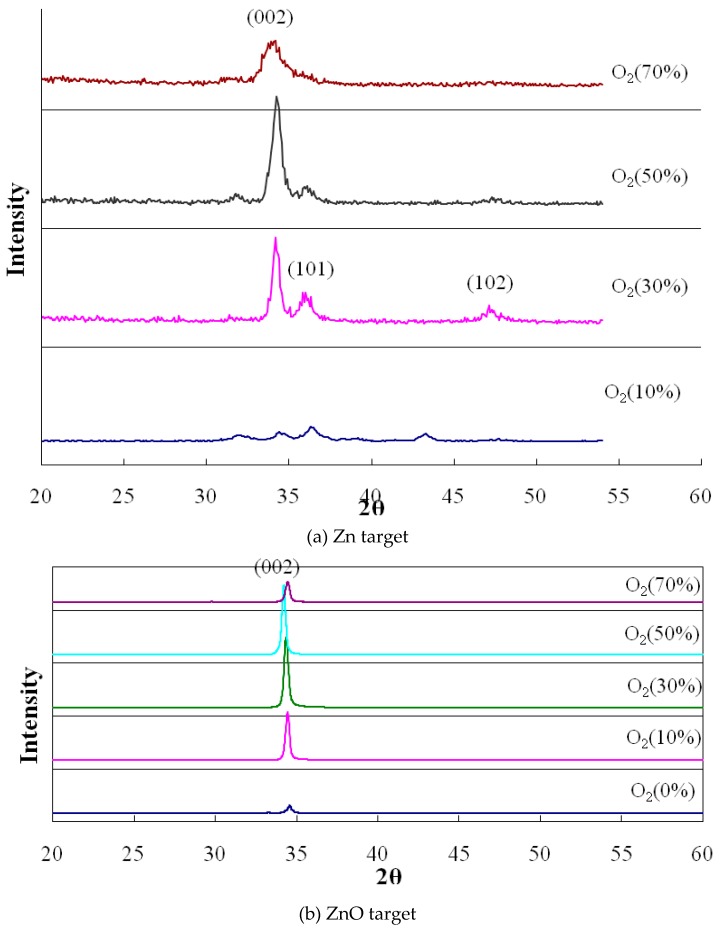
X-ray diffraction (XRD) diagram of the ZnO thin film for various amounts of oxygen for the targets (**a**) Zn and (**b**) ZnO.

The crystal array lattice in the deposited ZnO thin film was determined, in this study, by XRD (JEM-6500, JEOL, Akishima, Japan). Figure 8 indicates the XRD diagrams of the ZnO thin film for various amounts of oxygen by Zn and ZnO targets. The ZnO thin film was piezo-electric; its crystal direction needed a C axis by (002). The angle (2θ) of the greatest XRD diffraction strength was determined and crystal property was confirmed. The results demonstrate that the crystal array (002) of ZnO thin film was strongest at 2θ = 34°. The intensity was greatest (2θ angle) at 50% oxygen, for both targets. These results demonstrate that the ZnO thin film formed a better lattice array in 50% oxygen, as deposition of the thin film can complement oxygen atoms for a thin film in the sputtering reaction. Therefore, the array of crystalline particle was tidier. If too much oxygen gas was provided, the gas dissociation was reduced, reducing the plasma density. This phenomenon reduces the kinetic energy of atoms upon deposition and appears to make the crystal array less tidy.

### 3.5. Performance of SAW

**Figure 9:**
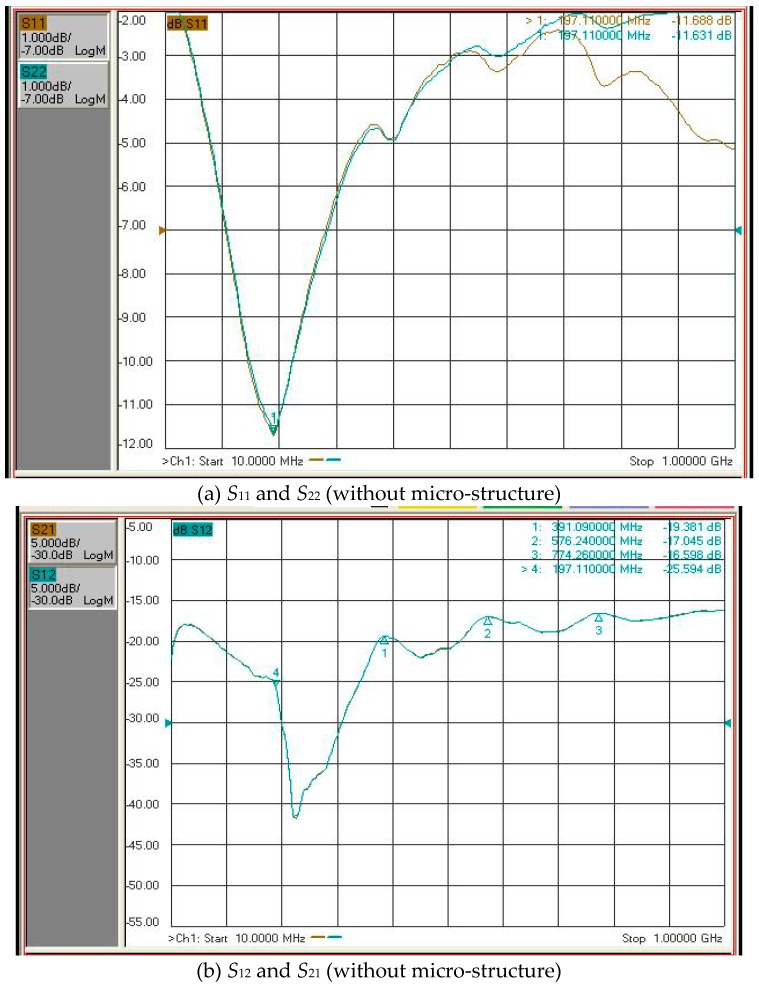

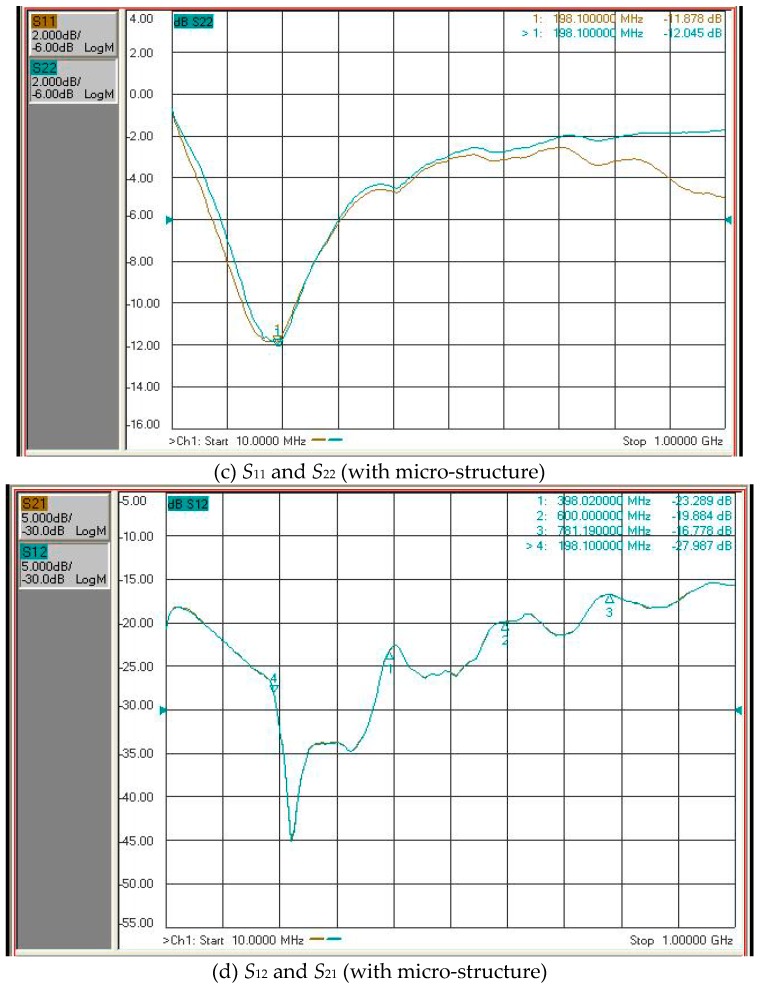
S-parameter diagram of the ZnO SAW with and without micro-structure, using 50% oxygen.

**Figure 10:**
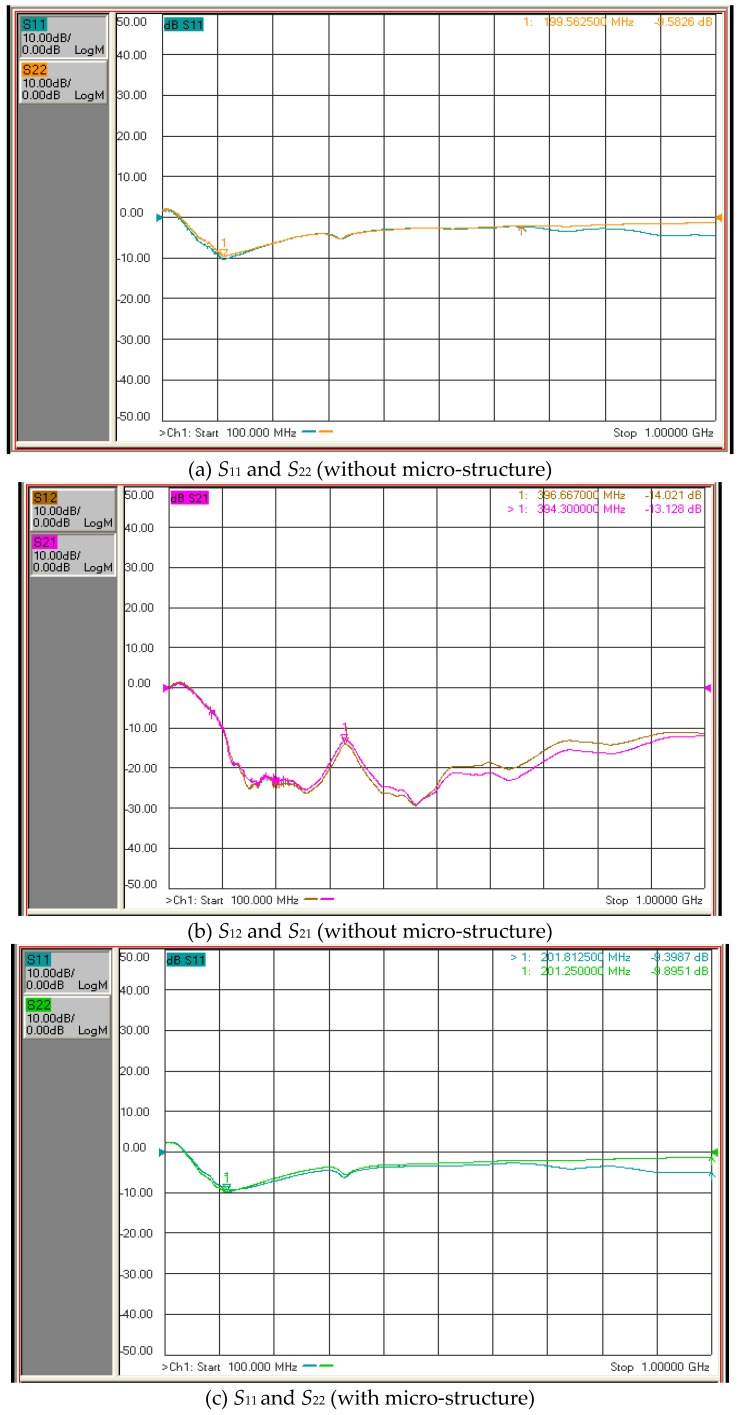

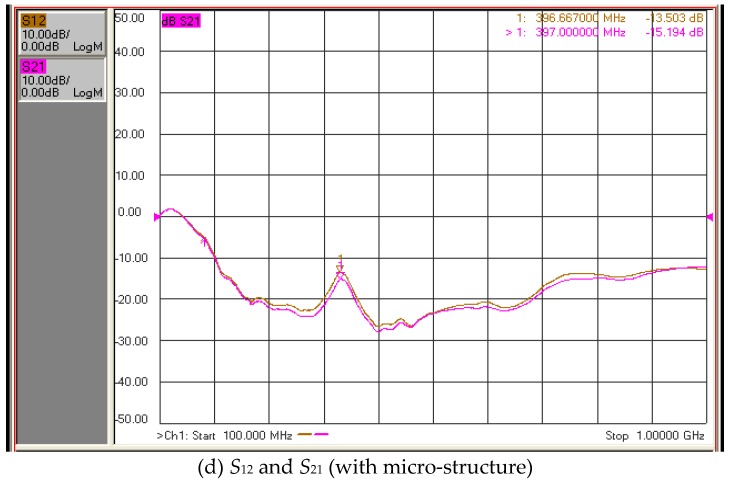
S-parameter diagram of ZnO SAW with and without micro-structure, using 30% oxygen.

The ZnO thin film had better material properties when the oxygen content was 30% or 50% and with the ZnO target. A network analyzer was employed to measure the performance of the SAW element. The scanning range of the network analyzer was 10 MHz–1 GHz. Figure 9a,b display the S-parameter diagrams of the ZnO SAW without micro-structure and using 50% oxygen. The results indicate that the maximum response frequency was 774 MHz and the insert loss was 16 dB. Figure 9c,d present the S-parameter diagram of the ZnO SAW with micro-structure and using 50% oxygen. The results reveal that the insertion loss was 16 dB and the maximum response frequency was 761 MHz. Figure 10 reveals the S-parameter diagram of ZnO SAW, with and without micro-structure, using 30% oxygen. The maximum response frequency was 394–396 MHz for the ZnO SAW without micro-structure and 396–397 MHz for the ZnO SAW with micro-structure. The insertion loss was 13 dB for the ZnO SAW without micro-structure and 13–15 dB for the ZnO SAW with micro-structure. The results indicate that the insertion loss of the ZnO thin film using 30% oxygen was less than that for 50% oxygen, while the surface roughness of the ZnO thin film using 50% oxygen was less than for 30% oxygen. The surface of the ZnO thin film using 50% oxygen exhibited ZnO crystal particles, according to SEM and AFM, increasing insertion loss. The insertion loss on the ZnO thin film for 30% oxygen was less than that for 50% oxygen. The signals *S*_11_ and *S*_22_ were very similar to each other and those of *S*_12_ and *S*_21_ were also similar, according to the S-parameter diagram, regardless of whether the ZnO SAW had micro-structure. These experimental results agree with the previous formula. The frequency paths of ZnO SAW, without and with micro-structure, were almost identical. Although the maximum response frequency of ZnO SAW without micro-structure was slightly different from that with micro-structure, the insertion losses between them were equal. In summary, the insertion loss did not increase upon including micro-structure into the SAW ZnO thin film. 

## 4. Conclusions

Surface acoustic wave devices are components that are in high demand in the sensor field. The deposition rate of ZnO thin film was highest on a Si substrate (94.8 nm/min) at 10% oxygen with a Zn target, followed by the deposition rates at 30%, 50%, and 70% oxygen. The deposition rate of ZnO thin film is highest on Si substrate (10.96 nm/min) at 0% oxygen applied with a ZnO target, followed by deposition rates at 10%, 30%, 50%, and 70% oxygen. The deposition rates of the ZnO thin film decreased on a Si substrate when the oxygen percentage increased for both Zn and ZnO targets. The deposition rate of thin film on a Si substrate using a Zn target exceeded that for the ZnO target. The surface roughness and surface morphology of the ZnO thin film by Zn target had optimal values when 50% oxygen was used. The same result was obtained for the ZnO target. The surface roughness and surface morphology of the ZnO thin film with the Zn target were less than those for the ZnO target. The lattice of crystal (002) of the ZnO thin film had optimal value when 50% oxygen was used, for both targets. The insertion loss of ZnO SAW using 30% oxygen was less than that using 50% oxygen. 

This experiment was successfully combined with the micro-electro-mechanical process, and surface acoustic wave components with unstructured structure and measurement area were successfully fabricated. Although the structural characteristics of the film were observed for differing oxygen content during film deposition, there are other parameters that could be used to adjust the film preparation, such as annealing temperature, power setting, substrate temperature, and so on, and introduce the parameters into Taguchi. The experimental method can definitely be used find a better-quality zinc oxide film. In this experiment, direct film sputtering was adopted. If multi-layer sputtering is used to prepare the surface acoustic wave element piezo-electric substrate, the surface acoustic wave element transmission rate and signal stability should increase.
